# Isocenter verification for linac‐based stereotactic radiation therapy: review of principles and techniques

**DOI:** 10.1120/jacmp.v12i4.3645

**Published:** 2011-11-15

**Authors:** Pejman Rowshanfarzad, Mahsheed Sabet, Daryl J. O'Connor, Peter B. Greer

**Affiliations:** ^1^ School of Mathematical and Physical Sciences University of Newcastle Newcastle NSW 2308; ^2^ Department of Radiation Oncology Calvary Mater Newcastle Hospital Newcastle NSW 2308 Australia

**Keywords:** isocenter verification, stereotactic radiosurgery, quality assurance, submillimeter accuracy

## Abstract

There have been several manual, semi‐automatic and fully‐automatic methods proposed for verification of the position of mechanical isocenter as part of comprehensive quality assurance programs required for linear accelerator‐based stereotactic radiosurgery/radiotherapy (SRS/SRT) treatments. In this paper, a systematic review has been carried out to discuss the present methods for isocenter verification and compare their characteristics, to help physicists in making a decision on selection of their quality assurance routine.

PACS numbers: 87.53.Ly, 87.56.Fc, 87.56.‐v

## I. INTRODUCTION

### A. Stereotactic radiosurgery/radiotherapy

The word stereotaxic or stereotactic is composed of the Greek word “stereos” meaning three dimensional and the Latin word “tactus” which means to touch. Stereotactic techniques are quite well known in various branches of neurosurgery.

Stereotactic radiosurgery is a highly‐precise technique for the delivery of high gradient conformal doses of ionizing radiation (usually from a linear accelerator) to a localized small target volume with typically 1–3 cm diameter.^(^ ^1^ ^–^ ^6^ ^)^ This method was invented by Lars Leksell, a Swedish neurosurgeon, as a noninvasive method to obliterate the brain tumors located in positions that are difficult to access for surgery.^(^ ^7^ ^)^ If the whole stereotactic dose is delivered in one session, the treatment is called stereotactic radiosurgery (SRS); if the stereotactic dose is delivered in multifraction sessions, it is known as stereotactic radiotherapy (SRT).

Stereotactic treatments may be delivered using three main methods Gamma Knife, X‐ray knife and CyberKnife. In a Gamma Knife unit, 201 Cobalt‐60 sources are arranged in a helmet pointing toward a target in the brain. The X‐ray knife technique is linac‐based and uses multiple noncoplanar arcs to aim at the center of the target positioned at the linac isocenter. The treatment beams may be shaped by micro multileaf collimators or solid circular cones. In both Gamma Knife and X‐ray knife methods, a stereotactic frame is used to fix the skull. Modern Cyber Knife techniques are frameless and utilize image guidance systems to localize the tumor (cranial or extracranial) and monitor its motion using X‐ray images of the bony anatomy. The gantry, which is a compact 6 MV linear accelerator mounted on a robotic arm, and the treatment table are moved to compensate for target motion.^(^ ^8^ ^)^

The X‐ray knife method for stereotactic treatments is more widely used, since it is cheaper, and the linac can also be used for other common radiotherapy treatments (such as intensity‐modulated radiation therapy).^(^ ^8^ ^)^ Therefore, the present work is focused on these linac‐based stereotactic systems.

The main advantage of stereotactic radiosurgery is minimum damage to the surrounding critical organs (usually brain tissue) by application of a collimated and confined beam directed toward a small preselected target lesion. This provides sharp dose falloff out of the target volume.^(^ ^2^ ^,^ ^4^ ^,^ ^5^ ^,^ ^9^ ^–^ ^14^ ^)^ As the whole treatment doses (e.g., about 20 Gy to a 5 mm area) are delivered in a single session during stereotactic radiosurgery treatments, it is important to have high spatial accuracy.^(^ ^5^ ^,^ ^13^ ^)^

Stereotactic radiotherapy requires repositioning of the patient in various fractions as closely as possible. This may introduce uncertainties to the treatment delivery. On the other hand, the time interval between fractions in this technique enables normal tissues to repair and thus improves the treatment outcome as a consequence of radiobiological effects.^(^ ^15^ ^–^ ^17^ ^)^

In order to deliver successful stereotactic radiosurgery/radiotherapy treatments, it is essential to know the linac isocenter position, which is taken as the beam primary reference location^(^ ^18^ ^,^ ^19^ ^)^ with submillimeter accuracy during several successive noncoplanar arcs, as well as its precise mechanical pattern during the gantry, collimator, and treatment table rotations.^(^ ^11^ ^,^ ^13^ ^,^ ^20^ ^–^ ^22^ ^)^

### B. Positional accuracy

For dose delivery techniques such as SRS or SRT, it is vitally important to ensure the accuracy of the treatment procedure.^(^ ^4^ ^,^ ^10^ ^,^ ^23^ ^)^ There are several sources of uncertainty in stereotactic treatment systems such as errors in patient positioning, target localization, and dose delivery.^(^ ^20^ ^,^ ^24^ ^)^ It is practically impossible to achieve perfect alignment mainly due to the presence of several geometric errors in the system.^(^ ^25^ ^)^ One of the critical geometric errors in SRS/SRT treatments is uncertainty in localizing the radiation field center,^(^ ^26^ ^,^ ^27^ ^)^ which directly affects the dosimetric accuracy^(^ ^28^ ^)^ and results in incorrect tumor targeting that may lead to the delivery of inadequate dose to the lesion and/or serious damage to the healthy adjacent tissues.^(^ ^25^ ^,^ ^29^ ^)^ Therefore, it is necessary to develop methods to reduce the probability of such errors by extensive and efficient quality assurance programs to ensure high‐level geometric accuracy of the treatment.^(^ ^13^ ^,^ ^30^ ^–^ ^32^ ^)^ This requires development of strict acceptance levels and safety margins by independent institutions.^(^ ^32^ ^–^ ^34^ ^)^

The most influential geometric characteristic of the SRS/SRT treatments is the exact position of the target relative to the linac mechanical isocenter during beam delivery.^(^ ^27^ ^,^ ^35^ ^,^ ^36^ ^)^ In ideal conditions, the mechanical isocenter is defined as the point of intersection of gantry, collimator, and treatment table rotation axes;^(^ ^1^ ^,^ ^18^ ^,^ ^37^ ^,^ ^38^ ^)^ however, with the rotation of gantry, treatment table, and collimator, the isocenter also moves in the space due to the mechanical limitations of the linac components.^(^ ^39^ ^–^ ^41^ ^)^ These limitations include gantry excursions during rotation due to its unbalanced weight which leads to bending or twisting of the gantry arm,^(^ ^13^ ^,^ ^30^ ^)^ and irregularities that mainly originate from the precision bearing system of movement control.^(^ ^1^ ^,^ ^13^ ^,^ ^30^ ^,^ ^42^ ^–^ ^47^ ^)^ The geometric position of the isocenter during rotations is usually assumed to be inside a virtual spherical volume. Minimizing the isocenter movement could improve the accuracy of stereotactic treatments^(^ ^18^ ^)^ and this issue has been considered with special attention. The AAPM Task Group Report 142 (2009) recommends that up to ±1 mm deviation between the radiation and mechanical isocenter is acceptable for SRS/SRT treatments.^(^ ^48^ ^)^ In cases where uncertainties in target position were less than 1 mm, minor effects on dose distribution have been observed which were reported as clinically unimportant;^(^ ^2^ ^,^ ^49^ ^)^ but discrepancies larger than 1 mm are not acceptable as they may lead to severe side effects and require adjustment of the relevant parts of the linac.^(^ ^1^ ^,^ ^50^ ^)^ It has been reported that 2 mm positioning error in spine SRS could lead to more than 5% loss of tumor coverage and more than 25% increase in dose delivery to the healthy tissues.^(^ ^51^ ^)^ With an accuracy of 1 mm for SRS treatments, the error in dose delivery was reduced to less than 2%.^(^ ^20^ ^)^ It must be noted that errors in SRS treatment delivery are nonrecoverable, since the treatment is delivered in one session, while there is a chance of compensation in multifraction treatments such as SRT. The isocenter verification process must be performed before each SRS/SRT treatment.^(^ ^36^ ^,^ ^52^ ^–^ ^55^ ^)^

At present a variety of methods are used for verification of the linac mechanical isocenter position. In this paper, these techniques are individually reviewed and discussed to make it easier for users to identify the points of strength and weakness in each, and to decide which method to choose for their isocenter quality assurance.

### C. Review of isocenter verification methods

The systematic search strategy through the indexed database “Pubmed”, “Scirus”, “Scopus” “GoogleScholar” and “ScienceDirect” was carried out using search terms including “isocenter verification”, “stereotactic quality assurance”, “stereotactic radiosurgery”, “isocenter offset”, “stereotactic quality control”, and “mechanical isocenter”. The cited references of these papers that were published in English before March 2011 have also been included.

## II. MATERIALS AND METHODS

### Methods Used for Mechanical Isocenter Verification

#### A. Mechanical pointer

The conventional method for isocenter verification in radiotherapy centers is to measure the distance between the tip of a mechanical pointer mounted on the gantry head and a fixed point mounted on the treatment table.^(^ ^1^ ^,^ ^22^ ^,^ ^30^ ^,^ ^39^ ^,^ ^56^ ^,^ ^57^ ^)^ This method is manual, laborious and time‐consuming, and the results depend on the human observer. It is also limited by the size of the tip of pointer which is not exactly a single point.^(^ ^31^ ^,^ ^45^ ^)^ Although in one study the point on the treatment table was defined by a sharp needle attached to a micrometer, for better accuracy the measurements were repeated at distinct angles with the gantry rotated clockwise and counterclockwise. The reproducibility of the method was 0.2 mm.^(^ ^19^ ^)^

It must be noted that mechanical pointers are susceptible to damage and can be easily dislocated; therefore, they are not suitable for routine use.^(^ ^49^ ^)^

#### B. Winston‐Lutz test

This technique was introduced by Lutz, Winston and Maleki at Harvard Medical School in 1988.^(^ ^1^ ^)^ The Winston‐Lutz (W‐L) phantom is a small metallic ball (made of steel, titanium or tungsten) that represents the planned target and is fixed on the treatment table by a locking mechanism. The phantom position is adjustable in three directions by means of a micrometer tool.^(^ ^1^ ^,^ ^31^ ^,^ ^39^ ^)^ The collimator used for SRS/SRT is attached to the gantry head and the ball is placed as closely as possible to the isocenter by aligning the marks on the phantom with the treatment room lasers. The collimated beam is used to expose the radiographic test film mounted perpendicular to the beam direction on a stand behind the ball. The difference between the center of the sphere shadow and the field center reveals the isocenter movement^(^ ^1^ ^)^ (which must be within ±1 mm for stereotactic treatments). Measurements should be repeated at cardinal angles (0°, 90°, 180°, and 270°), which requires changing the film for each setup.^(^ ^1^ ^,^ ^36^ ^)^ The offset is read on each film using transparent template guidance scales^(^ ^22^ ^,^ ^39^ ^,^ ^58^ ^)^ or scanning the film and software analysis.^(^ ^14^ ^,^ ^36^ ^)^ This method could be used to check the gantry, treatment table, and collimator in various angles.^(^ ^36^ ^,^ ^42^ ^,^ ^43^ ^)^

The Winston‐Lutz test was relatively simple^(^ ^2^ ^)^ and became quite popular,^(^ ^34^ ^,^ ^36^ ^)^ but it was based on films; therefore, it inherited all film‐related problems. The general disadvantages in using films include the cost of films, chemicals and processor maintenance, and occupation of archiving space. In addition, it is not possible to modify the properties of film images to improve contrast.^(^ ^23^ ^)^ Dust or marks on the film can lead to spikes in images.^(^ ^40^ ^)^ The film results for Winston‐Lutz test are not quantitative,^(^ ^26^ ^,^ ^52^ ^,^ ^59^ ^)^ and are based on manual evaluations^(^ ^24^ ^,^ ^34^ ^)^ and visual inspection — which makes them highly dependent on the skills of the observer.^(^ ^11^ ^,^ ^36^ ^,^ ^52^ ^,^ ^60^ ^)^ The uncertainty introduced by operator judgment has been reported to be 0.3 to 0.4 mm,^(^ ^36^ ^,^ ^61^ ^,^ ^62^ ^)^ although this could be reduced by scanning the films and using software analysis.^(^ ^14^ ^,^ ^36^ ^,^ ^58^ ^)^ In addition, film adjustment and processing are time‐consuming,^(^ ^11^ ^,^ ^36^ ^,^ ^52^ ^)^ which makes it difficult to perform the test before each treatment. Furthermore, the measurements made at discrete angles cannot perfectly represent the geometric status of the isocenter.^(^ ^63^ ^)^ The uncertainty in film measurements were reported to be ±0.3 mm by Lutz et al.,^(^ ^1^ ^)^ 0.2±0.1 mm by Friedman and Bova,^(^ ^43^ ^)^ and ±0.5 mm by Winkler et al.^(^ ^42^ ^)^ — which were relatively large regarding the ±1 mm acceptance criteria. Even in one study with GAFCHROMIC films, up to ±1 mm uncertainty was detected in isocenter positioning.^(^ ^64^ ^)^

A mathematical method was developed by Low et al.^(^ ^39^ ^)^ which used the film‐measured isocenter positional errors for eight gantry angle and couch settings to find the suitable offset for the phantom stand to minimize the distance between the linac isocenter and the target. A similar aim was followed by Grimm et al.,^(^ ^14^ ^)^ who developed an algorithm to reconstruct the W‐L phantom ball locus in three dimensions from two‐dimensional film images taken at certain couch and gantry angles and combined them with the images of lasers taken by digital cameras. The room lasers were repositioned for each angle to align with the isocenter using a special device that quickly gave the required displacements. The accuracy of the method was reported better than 0.25 mm for most cases.^(^ ^14^ ^)^

In another study on SRS/SRT treatment systems, seven film irradiations at different gantry and couch angles were used to determine the deviation of the beam and the target centers. A mathematical model was applied to analyze the data, which led to seven equations with five parameters. Solving the equations by two computer codes showed the direction and values of discrepancies which were then used for corrections. The accuracy of the procedure was tested by detection of the manually‐displaced target positions.^(^ ^57^ ^)^ The proposed method was too complicated and time‐consuming.^(^ ^27^ ^,^ ^28^ ^,^ ^50^ ^)^

The application of W‐L test has been extended to quality assurance procedures for proton therapy, where the acceptable range of isocenter position is even more limited, having been considered within ±0.5 mm accuracy by Ciangaru et al.^(^ ^40^ ^)^ In their study, GAFCHROMIC EBT films for 16 gantry angles were digitized; the image noise was reduced by subtraction of a blank film from the exposed film pixel by pixel and smoothed using an averaging MATLAB filter. But this method inherited all of the film‐related problems.

#### C. EPID‐based isocenter verification methods

Electronic portal imaging devices (EPIDs) gradually replaced films and made the isocenter verification test procedure much easier. EPIDs have many advantages over films such as the ability to re‐use, to provide digital images, to permit easy and quick data transfer, and to archive.^(^ ^23^ ^,^ ^37^ ^,^ ^52^ ^,^ ^58^ ^)^ The digital format of EPID images enables contrast enhancement^(^ ^65^ ^)^ which is very helpful in image analysis. It is important to note that EPIDs are currently included in the structure of modern linear accelerators and require no time‐consuming setup.^(^ ^63^ ^)^ The limitation of using EPIDs include the few combinations of gantry and couch angles where EPID images cannot be acquired.^(^ ^52^ ^)^

In the majority of QA procedures, the mechanical accuracy of the system is evaluated by using phantoms containing small, well‐defined markers at the center.^(^ ^31^ ^)^ The methods and algorithm designs for EPID‐based studies using W‐L phantom and some of the most popular phantoms are presented in the following section.

##### C.1 W‐L phantom

Many research groups have used EPIDs instead of films for the evaluation of discrepancies between the center of a W‐L phantom (target) and the linac mechanical isocenter. In one study, global thresholding technique was used to detect the center of circularly collimated radiation fields, while the center of the metallic sphere was determined by an algorithm applying bilinear interpolation and thus eliminating the observer error. The overall accuracy of the method was within ±0.2 mm. The processing took three minutes, which is much less than film‐based systems, and the method was tested for few angles.^(^ ^52^ ^)^

Global thresholding technique was also used in another study where the algorithm detected the center of the phantom and compared it with the center of square micro‐MLC–defined fields extracted from the penumbra profiles. The process took 15 minutes to complete and was tested for five different gantry and couch setups. The results were within 0.3 mm of film measurements.^(^ ^36^ ^)^ It must be noted that global thresholding technique is sensitive to noise.^(^ ^66^ ^)^

In a similar study by Torfeh et al.,^(^ ^21^ ^)^ thresholding method was used for finding the secondary collimator defined radiation field center, while the center of the sphere was determined by convolution of a Gaussian kernel with the EPID image. The method was applied to some distinct angles and tested by detection of an arbitrary phantom offset.^(^ ^21^ ^)^ The overall accuracy was not reported, and the sag in secondary collimator and EPID (which introduce inaccuracies) were not considered in the processing.

A different approach was used by Winey et al.,^(^ ^58^ ^)^ applying a double convolution method to the EPID image to find the center of the field and the ball separately with subpixel accuracy. Their work was based on a previously‐developed technique by Guizar‐Sicairos et al.^(^ ^67^ ^)^ The method was applied seven times at distinct angles and compared to measurements made by: (a) human observer using templates designed as a graphical user interface, and (b) an edge detection and center of mass algorithm. The proposed method was faster than both due to the higher speed of calculations in Fourier domain. It was also tested by applying known displacements to the phantom and detecting them using the algorithm. The accuracy was 0.1 mm.^(^ ^58^ ^)^

Calvo‐Ortega et al.^(^ ^62^ ^)^ reported the results of a package designed for the EPID‐based W‐L test. The field center was calculated from the penumbra of square profiles and the center of the ball was found using a summation filter. The method was tested by application of manual shifts to the phantom and detection of the displacements with the algorithm. It was also compared with film measurement results at five angles. The accuracy was reported up to 0.2 mm.^(^ ^62^ ^)^

In another study on W‐L test, the radiation field was defined by circular and rectangular collimators fixed to the gantry head. Images of the target at the isocenter were acquired (on EPID or EBT2 films) at discrete angles and analyzed:^(^ ^26^ ^)^ Sobel filter was first applied to each image to detect the edges of the field and the ball; then, Hough transform — which is quite popular in digital image processing — was implemented to localize both the radiation and the ball centers. The signal‐to‐noise ratio had a major effect on the accuracy of this method since Sobel and Hough operators are highly sensitive to noise.^(^ ^58^ ^,^ ^68^ ^–^ ^71^ ^)^ Sobel filter is also dependent on the object size^(^ ^69^ ^)^ and its output is almost always more than one value,^(^ ^68^ ^)^ which results in a thick detected edge and increases the uncertainty of results.^(^ ^58^ ^,^ ^72^ ^)^ It is worth mentioning that Hough transform prolongs the processing^(^ ^73^ ^,^ ^74^ ^)^ as it requires one second per image to process.^(^ ^26^ ^,^ ^58^ ^)^ However, the absolute error for this method was reported as 0.02 mm. Du et al.^(^ ^25^ ^)^ further investigated isocenter verification for SRS quality assurance by EPID‐based W‐L test in a number of MLC‐defined square fields at cardinal angles, using mechanical graticule. Similar to the previous work, Sobel filter and Hough transform operator were used to localize the centers of the radiation field and the ball bearing. A two‐component model was developed to overcome the issue of overlapping centers of the ball and graticule. A 0.47 mm systematic error was considered for the MLC leaf positioning. The problem with this method was sensitivity to variations in image intensity.^(^ ^25^ ^)^

A different image analysis method was introduced by Winkler et al.^(^ ^42^ ^)^ for EPID‐based W‐L test. The method was applied to jaw‐defined and circular collimator‐defined (used for SRS/ SRT) fields. EPID images were acquired at distinct angles and analyzed in the following steps: (a) segmenting the radiation field and locating its center, (b) segmenting the image of the tungsten sphere and locating its center, and finally (c) determining the deviation of the two centers. A convolution kernel was used to improve the sharpness of the ball image to enable more accurate edge detection by decreasing the noise. The segmentation required image magnification which introduced up to 0.02 mm error. However, the overall accuracy of the method was estimated by comparison to visual and radiographic film tests and was reported as 0.1 mm.^(^ ^42^ ^)^

##### C.2 Other phantoms

On Elekta Synergy linacs (Elekta, Stockholm, Sweden), the routine method for isocenter verification is to use a ball bearing phantom provided by the manufacturer, which has 8 mm diameter and is imaged at cardinal angles with the phantom aligned to the room lasers. The system software uses the edges of the jaw‐defined field and the center of ball bearing in each image. The field edges detected in images acquired at opposing angles were used to remove the jaw sag. This phantom was applied in one study as reference to benchmark the application of a QUASAR Penta‐Guide phantom (Modus Medical Devices Inc., London, Canada) where the central air cavity replaced the ball bearing.^(^ ^32^ ^)^ Canny filter was used along with a binary mask to detect the field edges. The lines defining the field edges were determined and Hough transform was applied to the image to identify the four corners of the square field. The field center was specified by finding the mean of the points at four corners. The center of the air cavity was determined from the EPID image by using a low‐pass filter to the center of the image (to reduce the noise) and applying thresholding technique to identify the air cavity edges. The center of the circle fitted to the results was the center of the air cavity. Comparison of the method with the standard ball bearing phantom results showed slight differences and the systematic error was less than 0.2 mm.^(^ ^32^ ^)^ The main concern about this method would be the application of Canny filter which results in broken edges.^(^ ^75^ ^)^ In addition, Hough transform method is sensitive to noise.^(^ ^71^ ^)^

QUASAR Penta‐Guide phantom has also been used with a simple voxel‐based contouring algorithm, considering the center of the contour as the isocenter. The results were compared with the isocenter indicated by the room lasers. The contouring algorithm involved a large amount of noise which introduced about 1 mm error to the outcome of the algorithm. The process took less than six minutes.^(^ ^31^ ^)^

A graphite cylindrical phantom provided by Varian Medical Systems (Varian Medical Systems, Palo Alto, CA) containing 16 tungsten carbide ball bearing markers was used in a different method by Mamalui‐Hunter et al.^(^ ^28^ ^)^ for isocenter verification. The geometric location of markers were used to assess the linac mechanical isocenter in addition to the gantry and couch angles, source‐to‐detector distance, couch vertical position, and gantry sag in each image. This method required the removal of nonuniform background using a numerical optimization function. The ball bearings were detected in the images using a Sobel filter‐based method. It was only reported for some distinct angles and the precision was better than 0.1 mm. This method has all the issues mentioned above for the application of Sobel edge detection filter.^(^ ^28^ ^)^

A more or less similar idea was used for a cubic phantom containing 13 steel ball bearings for the determination of a number of linac characteristics including the isocenter position from EPID images.^(^ ^76^ ^)^ The isocenter was found using 36 images (one per 10° gantry rotation) and fitting a curve through the estimated location of the radiation beam source. The location of the source was determined by giving weight to the signal intensity of each pixel. The center of the source trajectory was considered as the isocenter. The accuracy of the method was limited by the source penumbra, pixel noise, and uncertainty in measurements of the marker locations (which was partly corrected by using a global optimization algorithm). However, the accuracy of setting the ball bearings in the planned positions in phantom was reported 0.5 mm, which introduced some errors to the results. The accuracy of the method was reported up to 1.6 mm.^(^ ^76^ ^)^

Another phantom made of a radiopaque material containing nine spherical indicators was used by Sharpe et al.^(^ ^41^ ^)^ The central sphere was placed at the nominal isocenter set by the intersection of the lasers in the treatment room. EPID images were acquired with the radiation collimated through a jaw‐defined field at cardinal angles. The central axis of the field was determined from the field edges and compared with the target center of mass. The latter was averaged for opposing angles to remove the jaw flex. The results were noisy and the process took 12 minutes.^(^ ^41^ ^)^

#### D. Miscellaneous methods

**Direct monitoring:** In a proton therapy facility, isocenter deviations were directly measured by a CCD camera that monitored a scintillator screen exposed to a field containing a steel sphere at the isocenter.^(^ ^77^ ^)^ The ball shadow was detected inside the light spot generated by the collimator on the screen. An algorithm was used to first make circular fits through data points to find the contours of the beam and the ball shadows. Their centers were determined from the contoured edges. The accuracy of the method was examined by applying known offsets to the sphere position and detecting them by the algorithm, which was reported up to 0.25 mm and took 10 minutes to complete.^(^ ^77^ ^)^

**Computed radiography:** In a study based on Winston‐Lutz method, computed radiography images of a lead ball bearing were acquired at five combinations of couch and gantry angles in micro‐MLC–defined fields. The shifts in isocenter were manually determined on the magnified images.^(^ ^23^ ^)^ The accuracy of the method could be affected by the uncertainty in the position of micro‐MLC leaves.

**Optical methods:** Gibbs et al.^(^ ^78^ ^)^ investigated the mechanical stability of the isocenter using a diode laser mounted in the radiosurgery collimator. The laser was detected by a photodiode detector that had a position‐sensitive surface and rotated with the gantry to stay perpendicular to the laser beam at all times.^(^ ^78^ ^)^

**An optical tracking system** has been used by Skworcow et al.^(^ ^30^ ^)^ along with GAFCHROMIC films to find the mechanical isocenter. A mathematical optimization method was used to detect the isocenter position as well as the intersection locus of collimator axes at 24 gantry angles. The deviation of the two results was recorded as the isocenter displacement. This method was tested by applying manual offsets to the isocenter position and finding it with the proposed system, which showed up to 0.7 mm uncertainty.^(^ ^30^ ^)^

Brezovich et al.^(^ ^49^ ^)^ developed a method to investigate the wobble in the isocenter as a result of treatment table rotations. The gantry light field was limited by a circular aperture and a steel ball was set at the nominal isocenter. Light‐sensitive film images were taken with the table rotated to different angles and the thickness of the ring‐shaped image was used as a measure of the isocenter movements. The method was limited to nondynamic treatments and required 40 minutes to perform.^(^ ^49^ ^)^

**Polymer gel:** Another different approach for the evaluation of the isocenter in Leksell SRS/SRT system involved the application of polymer gels used for dosimetry.^(^ ^12^ ^)^ A head phantom including a polymer gel vessel at the isocenter area was irradiated using a collimated treatment field. The centre of the 50% dose level was considered as the isocenter and compared with the treatment planning system. It must be noted that the application of gel introduced errors due to uncertainties in dose determination (up to 3% for the high‐dose area). The noise in magnetic resonance evaluation images could affect the data, particularly in the point of normalization which would deteriorate the whole profile.^(^ ^12^ ^)^ Furthermore, this method requires a long time for preparation, processing, and analysis of data, making it impractical for routine clinical application.

**Digital micrometer:** In one study, the deviation of the treatment table axis from the isocenter was measured along with the angle between the table axis and vertical direction using a two‐dimensional digital micrometer.^(^ ^50^ ^)^ Four angles were examined, and the mean axis position was calculated with a standard deviation which was attributed to the uncertainty in the alignment of lasers and the table wobble during rotation. Loading the table did not show a considerable effect on the table wobble.^(^ ^50^ ^)^

## III. CONCLUSIONS

The issue of isocenter verification with a high degree of spatial accuracy is extremely important in SRS/SRT treatments and requires highly‐efficient quality assurance scheduling. In this paper, various techniques and algorithms suggested by different groups of researchers leading to improved accuracy for verification of the mechanical linac isocenter have been summarized and discussed to make it easier for the physicists to decide which method to choose for their routine quality assurance procedure.

The problems with conventional methods of isocenter verification, including the use of mechanical pointer and film‐based Winston‐Lutz method, are described in detail in the introduction section and briefly listed in Table 1. As a result, there has been a growing interest on developing techniques to automatically investigate the stability of the isocenter position with submillimeter accuracy.

**Table 1:** Comparison of the three main methods for mechanical isocenter verification.

*Method*	*Advantages*	*Disadvantages*
Mechanical pointer	Does not require programming or special devices.	Manual, laborious. Time‐consuming. Observer‐dependent. Large measurement uncertainties.
Film based W‐L test	Better accuracy than pointer. Easy phantom setup. Semi‐automatic.	Only few angles can be tested. Requires film setup and processing. Nondigital data. No contrast enhancement. Observer‐dependent.
EPID based W‐L test	Digital images. Quick setup. Contrast enhancement. Automatic. Require lower radiation than films.	Impossible at certain gantry and couch angles. Lower resolution than films.

Several algorithms have been proposed for automatic isocenter verification that are mainly based on using EPID images of a phantom for the assessments, although there are a few other less popular techniques using different methods or detectors. All EPID‐based techniques have the advantages of being quick and automatic, providing digital images with no major setup required for the imaging device, and producing quantitative results for the isocenter offset. The results can be used to adjust the lasers or mechanical features involved in the gantry or couch rotations. The advantages and drawbacks of each EPID‐based algorithm are summarized in Table 2.

**Table 2:** Comparison of various EPID‐based algorithms for isocenter verification.

*EPID‐based Algorithms*	*Phantom*	*Advantages*	*Disadvantages*
Global thresholding technique^(^ ^20^ ^,^ ^35^ ^,^ ^51^ ^)^	W‐L	Simple. Up to 0.3 mm accuracy.	Sensitive to noise. Needs further processing to achieve subpixel accuracy.
Double convolution method^(^ ^57^ ^)^	W‐L	Fast. 0.1 mm accuracy.	Complicated method
Sobel edge detection filter+Hough transform ^(^ ^24^ ^,^ ^25^ ^)^	W‐L	0.02 mm accuracy.	Relatively slow. Sensitive to noise and object size. Increased uncertainty due to detection of thick edges.
Segmentation+convolution kernel ^(^ ^41^ ^)^	W‐L	Simple. 0.1 mm accuracy	Image magnification introduces errors. Low resolution in radiation field segmentation.
Canny edge detection filter+Hough transform ^(^ ^31^ ^)^	QUASAR	0.2 mm accuracy	Relatively slow. Broken edges. Sensitive to noise.
Contouring algorithm^(^ ^30^ ^)^	QUASAR	Subpixel accuracy	Slow. Large uncertainties.
Sobel edge detection filter+numerical optimization ^(^ ^27^ ^)^	Varian	0.1 mm accuracy	Sensitive to noise and object size. Increased uncertainty due to detection of thick edges.
Edge detection filter+target center of mass ^(^ ^40^ ^)^	In‐house designed	Submillimeter accuracy	Long processing times. Noisy results.
Signal intensity weighting+global optimization ^(^ ^75^ ^)^	In‐house designed	None	Complicated. Uncertainties due to source penumbra and pixel noise. 1.6 mm accuracy.

In conclusion, the basic methods for automatic investigation of the position of linac mechanical isocenter at some gantry and couch angles have been established. Nevertheless, there is still room for improvement of the algorithms. Future research can lead to the development of faster and more accurate methods for the determination of gantry or couch wobbles during SRS/SRT treatments.

## ACKNOWLEDGMENTS

This work was supported by the NHMRC (Grant No. 569211). One of the authors (PR) gratefully acknowledges the award of the UNIPRS scholarship from the University of Newcastle.
